# Biochemical and microbiological analysis of the saliva of institutionalized elderly: With edentulism, use of dentures and presence of biofilm

**DOI:** 10.4317/jced.56842

**Published:** 2020-07-01

**Authors:** Luiza A. S. Montenegro, Ilky-Pollansky Silva e Farias, Elza-Cristina-Farias de Araújo, Jannerson-César-Xavier de Pontes, Maria-Letícia-Barbosa Raymundo, Silmone-Alves de Sousa, Leopoldina F. D. Almeida, Yuri-Wanderley Cavalcanti

**Affiliations:** 1 Clinical and Social Dentistry Department. Federal University of Paraiba. Joao Pessoa, PB, Brazil

## Abstract

**Background:**

To analyze biochemical and microbiological parameters of the saliva of institutionalized elders and to investigate the relation of these parameters with edentulism, use of dentures and presence of biofilm.

**Material and Methods:**

A cross-sectional study carried was out in seven long-term institutions. Samples (n=161) of unstimulated saliva were collected for analysis of salivary flow, quantification of total proteins and identification of microorganisms. Oral examination was carried out to verify the number of missing teeth, the use of dentures and the presence of visible biofilm on the surface of teeth and dentures. Associations were performed using chi-square or Fisher’s exact test (α<0.05). Mann-Whitney Test was used to identify differences in the salivary flow and total proteins (α<0.05).

**Results:**

There was no association between the type of edentulism and use of dentures with biochemical and microbiological parameters of saliva (*p*>0.05). Associations were observed between the presence of dentures biofilm and the colonization of *Streptococcus* sp. (*p*=0.038) and *Candida* sp. (*p*=0.03).

**Conclusions:**

The absence of teeth and use of dentures do not influence the amount of total proteins and the microorganisms count in saliva. Denture biofilms are associated with the presence of *Streptococcus* sp. and *Candida* sp. in saliva of institutionalized elders.

** Key words:**Candida, long-stay institutions for elders, saliva, Streptococcus, Staphylococcus.

## Introduction

The physiological and morphological changes that accompany advancing age make older people more likely to become ill (1,2). These changes when associated with prolonged use of medications may potentiate or cause systemic imbalances. In the oral cavity, the decrease in salivary volume and the change in saliva composition may be the consequence of systemic diseases or adverse drug side effects (3,4). Salivary secretions are important for oral health, exerting protective activity against oral infectious diseases. Decreased salivary flow may increase the risk of candidiasis, caries susceptibility, periodontal disease and tooth loss (5).

Elders residents in long-stay institutions for the elderly (LSIE) are more likely to acquire diseases and consequently to use drug therapies. The unfavorable social conditions of this population, when compared to those who live in the family environment, influence their general health, making them more susceptible to illness (6-9).

Profile characteristics of institutionalized elderly such as edentulism and the use of dental prostheses may cause the presence of oral diseases. These changes may imply oral microbiota imbalance, resulting in biofilm proliferation and possible opportunistic infections (10).

Opportunistic fungal infections of the *Candida* sp. genus have a high prevalence and morbidity in debilitated individuals and may spread into the bloodstream and result in a risk of mortality. Excessive proliferation of *Candida* sp. is responsible for the development of candidiasis, which is often associated with the use of removable prostheses and systemic disorders such as diabetes mellitus (11-13).

In addition to fungal colonization, the change in salivary composition may influence the increase in the number of *Streptococcus* and *Staphylococcus* sp. *Streptococcus* microorganisms are primarily related to biofilm and dental caries development, but their excessive proliferation may be a risk factor for the development or aggravation of systemic cardiovascular diseases (14). Other pathogens, such as Staphylococcus sp. that are present in the respiratory tract, and in gastrointestinal and skin infections, may colonize the oral cavity in association with oral etiology changes, such as caries disease and periodontal disease in individuals with some systemic imbalance (15-17).

Consequently, elderly residents of LSIE may have their health condition aggravated by opportunistic infections of the oral cavity. However, there is limited knowledge about salivary parameters and oral microbiota of institutionalized elderly, as well as their relationship with edentulism, use of dentures and presence of biofilm.

Thus, this study aims to analyze the saliva of institutionalized elderly regarding salivary flow, amount of total protein and colonization by *Streptococcus* sp., *Staphylococcus* sp. and *Candida* sp. These parameters were related to the type of edentulism (partial or total), use of dentures and presence of visible biofilm in teeth and dentures.

## Material and Methods

-Study Design

A cross-sectional, inductive approach study with a comparative-statistical and descriptive-statistical procedure was conducted using direct documentation techniques and extensive direct observation (18). The study was conducted with elderly people in long-term care facilities in the metropolitan region of João Pessoa from July 2018 to December 2018.

-Ethical Aspects

This research was approved by the Research Ethics Committee of the Health Sciences Center of the Federal University of Paraíba (CAAE research protocol: 66122917.6.0000.5188). The ethical standards of the National Research Committee were met, as well as the Helsinki Declaration of 1964 and its subsequent amendments. All participants provided written informed consent.

-Volunteers and sampling

Seven regularly accredited long-stay institutions for the elderly (LSIE) were included in the metropolitan region of the city of João Pessoa-PB, in which allowed authorization to conduct the research.

The researchers invited the elderly residents, being free to choose whether to participate in this research. Elderly people with severe dementia or in a limiting condition for the study (inability to perform oral examinations or answer the questionnaires) were excluded from the sample.

The institutionalized elderly population in the metropolitan region of João Pessoa is made up of 398 individuals. However, the participation rate was estimated at around 40%, since many of the institutionalized individuals did not benefit from full exercise of their cognitive functions. Thus, 193 eligible institutionalized elderly were determined for the study, of which 161 performed the oral examination and saliva collection.

-Oral Examination

The oral examination evaluated the presence of teeth in the mouth, the need for dental prostheses and the presence of visible biofilm in teeth and dental prostheses. The examinations were performed by trained examiners using personal protective equipment (PPE), in the environment provided by long-term care facilities, under natural light, with the elderly sitting in a comfortable position. The individuals were categorized as partial or total edentulous (absence of all teeth), as well as regarding the use of dental prostheses.

-Saliva Collection

The collection was performed in a private environment and participants were instructed to remain resting for 2 minutes. All the accumulated saliva content was expelled into a polypropylene tube. Saliva samples were conditioned at low temperatures (6-10 °C) for further processing. 

-Salivary Flow and Quantification of Total Proteins in Saliva

Participants’ saliva samples were measured for volume (mL) to determine the amount of unstimulated saliva (ratio of volume of saliva produced per unit of time). Subjects were categorized into normal salivary flow (greater than 0.2 ml / min), low flow (0.1-0.19 ml / min) and hyposalivation (less than 0.1 ml / min) (19).

To quantify total proteins, saliva samples (20 μL) were homogenized in Biuret reagent (20 μL) and submitted for reading in a spectrophotometer (545nm).

-Quantification of *Streptococcus*, *Staphylococcus* and *Candida*

Saliva microorganisms were quantified by sowing saliva samples (20 μL) in presumptive identification culture medium. Streptococci of the mitis mutans subgroup (*Streptococcus* sp.) were seeded on Mitis Salivarius Agar (MSA) medium with 15% sucrose. Staphylococcus sp. were identified in Agar Sal Mannitol culture medium. For *Candida* sp. quantification, the culture medium Saburaud Dextrose Agar (ASD) added with chlorophenicol was used.

 After sowing, the plates were cultivated at 35-37 ºC, for 24 h, under aerobic atmosphere. After this period, the presence and absence of colony forming units (CFU) was observed.

-Data Analysis

Data were organized in an Excel® spreadsheet and then transferred to the IBM Statistical Package for Social Sciences software (IBM SPSS, v. 20, Chicago, IL) for statistical analysis.

Associations between the presence of microorganisms in saliva with edentulism, the use of dentures and the presence of visible biofilm in teeth and dentures were performed using Chi-square or Fisher’s exact test. Differences in the saliva’s biochemical parameters (salivary flow and total proteins) according to edentulism, use or not of dentures and presence of visible biofilm in teeth and dental prostheses were investigated by the Mann-Whitney Test (α <0.05).

## Results

Among the total of investigated elders (n = 161), 39.1% (n = 61) were partially edentulous and 60.9% (n = 95) totally edentulous. Of the total, 48.7% (n = 77) were users of dentures.

According to the presence of microorganism in the saliva, the genus *Staphylococcus* sp. was present in 14.1% (n = 22) of patients with total edentulism and 13.5% (n = 21) with partial edentulism. As for the use of dentures, this microorganism was present in 11.4% (n = 18) of the elders who did not use prostheses and 15.8% (n = 25) present in users (Table 1).

**Table 1:**
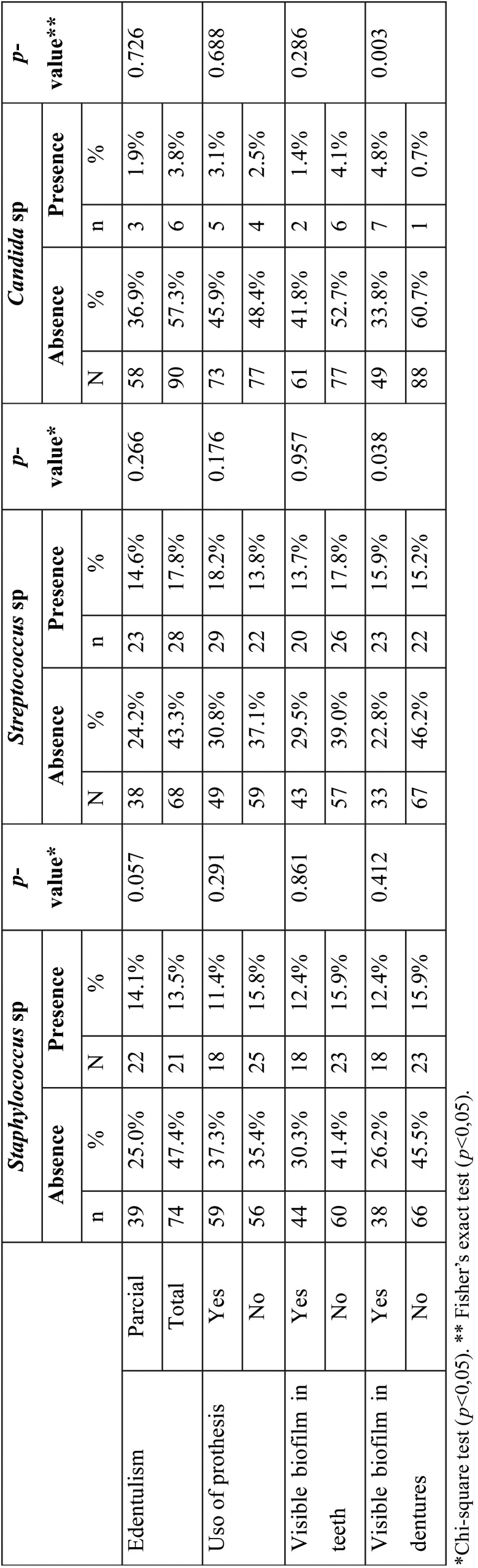
Colonization by *Staphylococcus* sp, *Streptococcus* sp, *Candida* sp according to edentulism and the use of dentures.

The presence of the genus *Streptococcus* sp. was found in 14.6% (n = 23) of individuals with total edentulism and 17.8% (n = 28) with partial edentulism. Regarding the use of dentures, the presence of *Streptococcus* sp. was observed in 18.2% (n = 29) of non-users and 13.8% (n = 22) in denture wearers (Table 1).

The presence of the genus *Candida* sp. was observed in 1.9% (n = 3) of elderly people with total edentulism and 3.8% (n = 6) with partial edentulism. Those who were not dentures users and presented *Candida* sp. corresponded to 3.1% (n = 5) and dentures users totaled 2.5% (n = 4) (Table 1).

According to the type of edentulism (partial and total) and the use of dentures, there was no significant association (*p*> 0.05) with the presence of these microorganisms (*Staphylococcus* sp., *Streptococcus* sp., *Candida* sp.). The presence of visible biofilm in prostheses showed a statistically significant association with the presence of Streptococcus sp. and *Candida* sp. in the saliva of the elders that were analyzed (*p* <0.05) (Table 1).

When analyzing the biochemical parameters of saliva, unstimulated salivary flow (ml/min) in the elderly with partial edentulism corresponded to a median of 0.23 ml/min and in the elderly with total edentulism, corresponded to 0.25 ml/min. According to the use of dentures, the unstimulated salivary flow corresponded to 0.25 ml/min (Table 2).

**Table 2:**
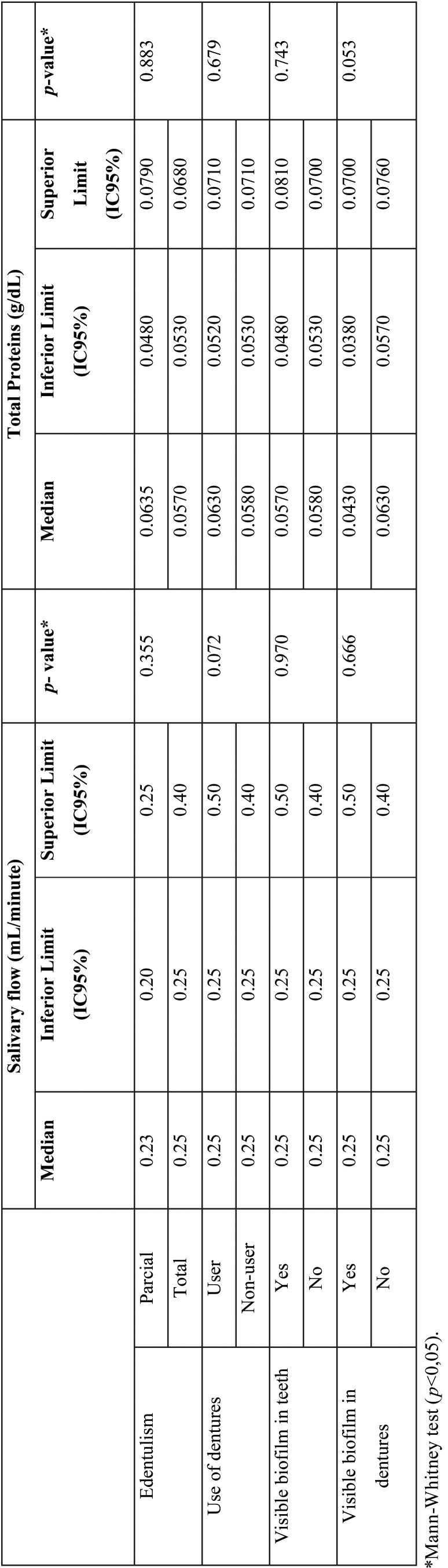
Salivary parameters according to edentulism and use of dentures.

Total saliva proteins (g/dL) in the elderly with partial edentulism corresponded to a median of 0.0570 (g/dL) and those classified with partial edentulism corresponded to 0.0635 (g/dL). According to the use of prostheses, those who did not use them had a concentration of 0.0630 (g/dL), and those who did, 0.0580 (g/dL) (Table 2).

There was no difference in the amount of total protein in saliva and the volume of saliva flow between the type of edentulism (partial or total) and use of dentures (*p*>0.05) (Table 2).

## Discussion

Hyposalivation can be attributed to morphophysiological changes resulting from the aging process (20). In addition, the reduction in salivary flow may be attributed to the side effects and adverse effects of drug therapies for the treatment of chronic diseases (21-23). In this study, elderly people with normal salivary flow were observed.

There were no associations between edentulism, dentures use and salivary flow variables, since it is a factor generally determined by systemic, rather than local conditions. The presence of visible biofilm in teeth or prostheses was not associated with salivary flow. Decreasing the amount of saliva reduces self-cleaning capacity, compromising oral hygiene and changes in oral microflora and salivary composition (24). However, according to our results, although the literature attributes saliva to the mechanical cleaning factor in the oral cavity, brushing remains a major factor in biofilm control for the institutionalized elderly population.

Human saliva is composed of several components, including proteins related to the defense and immune response of the individual. Protein composition changes according to gender, age and presence of systemic diseases (25). However, in our analysis there were no associations between the amount of total protein in the saliva and the presence of visible biofilm in teeth and dentures.

All the elderly had some type of edentulism, with predominance of total edentulous. However, even with indication for prosthetic rehabilitation, 51.2% of the elders did not use any type of dentures. We observed the absence of association between the edentulism variables, use of dentures, and microbiological parameters (*Staphylococcus* sp. *Streptococcus* sp., *Candida* sp.). Since most of the elderly were total edentulous and not wearing prostheses, this justifies the low prevalence in the oral cavity.

There was no association found in this study between edentulism, use of dentures and presence of biofilm with the colonization of *Staphylococcus* sp. Microorganisms of this genus commonly colonize the upper airways and are uncommon in the dental biofilm. The presence of this microorganism in the oral microbiota may contribute to increase the prevalence of systemic diseases, such as aspiration pneumonia (26-28).

Among the elderly who had visible prosthetic biofilm, there was an association with colonization of *Streptococcus* sp. Excessive colonization of this microorganism and the consequent dissemination in the bloodstream can result in cardiovascular problems such as atheroma calcification and bacterial endocarditis (29,30).

The profile of institutionalized elders in Brazil is of individuals with systemic diseases, considered fragile due to the socioeconomic and demographic context in which they are inserted (30-32). Thus, the need to control infections in the oral cavity since institutionalized elderly people have more risks of systemic diseases.

Association was observed between the presence of biofilm in dentures and *Candida* sp. A comparative study between institutionalized and non-institutionalized elderly individuals observed higher fungal colonization and mucosal lesions in elderly residents of long-term care institutions (33,34). Therefore, measures should be taken to favor the oral hygiene of these individuals and their prostheses.

The results of this study are representative for elderly people without a higher degree of cognitive disorder and living in long-term institutions in metropolitan regions of northeastern Brazilian capitals. They should be interpreted with caution due to the cross-sectional design employed. Future studies should consider a multivariate model that relates the biochemical and microbiological parameters of saliva.

## Conclusions

The presence of visible biofilm in dentures is associated with the presence of *Streptococcus* sp. and *Candida* sp. in the saliva of the institutionalized elderly people. The use of dentures and edentulism do not influence the biochemical and microbiological parameters of saliva.
